# A Systematic Review Investigating Healthy Lifestyle Interventions Incorporating Goal Setting Strategies for Preventing Excess Gestational Weight Gain

**DOI:** 10.1371/journal.pone.0039503

**Published:** 2012-07-05

**Authors:** Mary Jane Brown, Marlene Sinclair, Dianne Liddle, Alyson J. Hill, Elaine Madden, Janine Stockdale

**Affiliations:** 1 Institute of Nursing Research, University of Ulster, Newtownabbey, Northern Ireland; 2 School of Health Sciences, University of Ulster, Newtownabbey, Northern Ireland; 3 School of Biomedical Sciences, University of Ulster, Coleraine, Northern Ireland; 4 Head of Midwifery and Gynaecology, South Eastern Health and Social Care Trust, Belfast, Northern Ireland; 5 School of Nursing and Midwifery, Trinity College Dublin, Dublin, Ireland; University of Colorado Denver, United States of America

## Abstract

**Background:**

Excess gestational weight gain (GWG) is an important risk factor for long term obesity in women. However, current interventions aimed at preventing excess GWG appear to have a limited effect. Several studies have highlighted the importance of linking theory with empirical evidence for producing effective interventions for behaviour change. Theorists have demonstrated that goals can be an important source of human motivation and goal setting has shown promise in promoting diet and physical activity behaviour change within non-pregnant individuals. The use of goal setting as a behaviour change strategy has been systematically evaluated within overweight and obese individuals, yet its use within pregnancy has not yet been systematically explored.

**Aim of review:**

To explore the use of goal setting within healthy lifestyle interventions for the prevention of excess GWG.

**Data collection and analysis:**

Searches were conducted in seven databases alongside hand searching of relevant journals and citation tracking. Studies were included if interventions used goal setting alongside modification of diet and/or physical activity with an aim to prevent excess GWG. The PRISMA guidelines were followed and a two-stage methodological approach was used. Stage one focused on systematically evaluating the methodological quality of included interventions. The second stage assessed intervention integrity and the implementation of key goal setting components.

**Findings:**

From a total of 839 citations, 54 full-text articles were assessed for eligibility and 5 studies met the inclusion criteria. Among interventions reporting positive results a combination of individualised diet and physical activity goals, self-monitoring and performance feedback indicators were described as active components.

**Conclusion:**

Interventions based on goal setting appear to be useful for helping women achieve optimal weight gain during pregnancy. However, overweight and obese women may require more theoretically-designed interventions. Further high quality, theoretically-designed interventions are required to determine the most effective and replicable components for optimal GWG.

## Introduction

A recent report by the Centre for Maternal and Child Enquiries, identified 38,478 women with a BMI (body mass index) ≥35 (class II and class III obesity) gave birth every year in the United Kingdom (UK) [1]. Overweight and obesity in pregnancy are associated with adverse maternal and perinatal outcomes; these include an increased risk of post term delivery, gestational diabetes, pre-eclampsia, miscarriage and stillbirth [2]–[4]. In addition, maternal obesity and being overweight was found to be a contributing factor in more than 50% of maternal deaths between 2003 and 2005 [5].

Evidence suggests that there are two important factors that contribute to the long term development of overweight and obesity in women; excess weight gain during pregnancy and failure to lose excess weight after pregnancy [6]–[8]. There are no official UK recommendations for optimal weight gain during pregnancy but the Institute of Medicine (IoM) in the United States of America (USA) have recommended a weight gain range during pregnancy based on women’s pre-pregnancy BMI [9]. Current guidelines from the National Institute for Health and Clinical Excellence (NICE) recommend that all women, regardless of BMI should be provided with information and advice on diet and physical activity early on in their pregnancy [10]. In addition, the National Health Service (NHS) Pregnancy Planner provides helpful information about diet and exercise in pregnancy but information alone is often insufficient to bring about a change in behaviour [11]. Facilitating women to gain optimal weight during pregnancy could therefore prevent them from gaining and retaining excess gestational and postpartum weight [12].

Pregnancy is thought to be a “teachable” period that can have positive, long term outcomes [13]. It is also recognised as an appropriate opportunity to address rising levels of obesity and initiate behaviour change through the implementation of educational programs for weight management [10]–[14]. Phelan [13] suggests that the concern women have for the health of their unborn baby can provide significant motivation in itself to promote lifestyle changes.

Recent reviews of weight management interventions in pregnancy have reported mixed results. Two reviews performed a meta-analysis, to determine the effect of healthy lifestyle interventions for reducing excess gestational weight gain (GWG), and both found the interventions to be successful [15], [16]. However, Campbell et al. [17] conducted a meta-analysis of controlled trials together with a thematic synthesis of qualitative studies and reported no significant difference in GWG amongst participants in the intervention group compared with the control group. Moderate to high heterogeneity was reported in all three studies in relation to study designs, participants, interventions and outcomes.

A number of published reviews have sought to identify and evaluate key variables for weight management in pregnancy (e.g. diet and physical activity) [15], [18]–[22] again with varied results. Streuling et al. [15] concluded that interventions combining physical activity with diet counselling and additional weight monitoring strategies were successful for obtaining and sustaining optimal weight. Two subsequent reviews [19], [20] indicated that lower energy intake or physical activity may help prevent excess GWG. In reviews by Skouteris et al. [18] and Dodd et al. [21], evidence was inconclusive and the effect of modifying diet and physical activity for weight management in pregnancy was unclear.

A current lack of well-designed interventions of strong methodological quality has hindered the development of evidence-based recommendations for clinical practice [23]. Current interventions show limited effectiveness, and have been criticised for poor methodological quality, lack of theoretical frameworks, and high attrition rates [15], [24]–[26]. Therefore, a different approach is needed to determine the most effective interventions for weight management in pregnancy.

### Rationale

Past reviews have focused mainly on identifying key variables and their effectiveness in preventing excess GWG. Little attention has been given to the role of theory in producing effective interventions of clinical relevance. Whether or not theory contributes to the effectiveness of interventions is uncertain [16], [27]. However, a report by Green [28] and more recent guidelines from the Medical Research Council [29] highlight the importance of linking theory with empirical evidence in the context of evidence-based practice.

Theorists have determined that goals can be an important source of human motivation. Goal intention and goal setting have shown promise in promoting dietary and physical activity behaviour change among non-pregnant adults [30]–[32]. The recent review by Gardner et al. [16] identified goal setting and the associated self-monitoring and provision of feedback on performance as the most commonly used behaviour change techniques within these types of interventions.

The use of goal setting as a behaviour change strategy has been systematically evaluated within overweight and obese individuals [32], yet its use within pregnancy has not yet been systematically explored.

### Aim of Review

To explore the use of goal setting within healthy lifestyle interventions for the prevention of excess GWG.

### Objectives

To assess the methodological quality of interventions to prevent excess GWG.

To investigate the integrity of these interventions.

To explore how goal setting is used within these interventions and related outcomes.

## Methods

### Overview

The PRISMA guidelines [33] (see figure S1) and the Cochrane Collaboration Guidelines for Systematic Reviews of Health Promotion and Public Health Interventions [34] were used as a methodological template for this review. A two-stage methodological approach was used:

The first stage focused on systematically evaluating the methodological quality and validity of included interventions.

The second stage assessed intervention integrity and implementation: assessment of five different dimensions of integrity [35] and the application of additional goal setting components.

### Inclusion Criteria

The following inclusion criteria were applied:

#### Types of participants

The study population included healthy pregnant women who were ≥18 years old.

#### Types of intervention

Interventions using goal setting (either explicitly or non-explicitly) alongside modification to diet and physical activity/exercise levels (alone or in combination) with an aim to prevent excess GWG.

#### Types of outcomes

Primary Outcomes – GWG was considered an important primary outcome as an indicator of underlying motivation.

Secondary outcomes –To evaluate the motivational effects of goal setting interventions tested, psycho-social outcomes such as maternal self-efficacy were also explored.

#### Study design

Only randomised controlled trials (RCTs) of strong or moderate methodological quality were selected. RCTs are regarded as the most powerful method of determining cause-effect relationships between phenomena [36].

### Exclusion Criteria

Studies having only their abstracts available were excluded, as sufficient detail is needed to identify any non-explicit use of goal setting. Studies aimed at modifying diet and/or exercise during pregnancy for the primary purpose of improving or managing a specific disease (e.g. gestational diabetes) were also excluded. Finally, studies that focused on teenage mothers were excluded as evidence demonstrates that teenagers behave differently and experience different motivational barriers than other mothers [37], [38].

### Search Strategy

**Figure 1:**
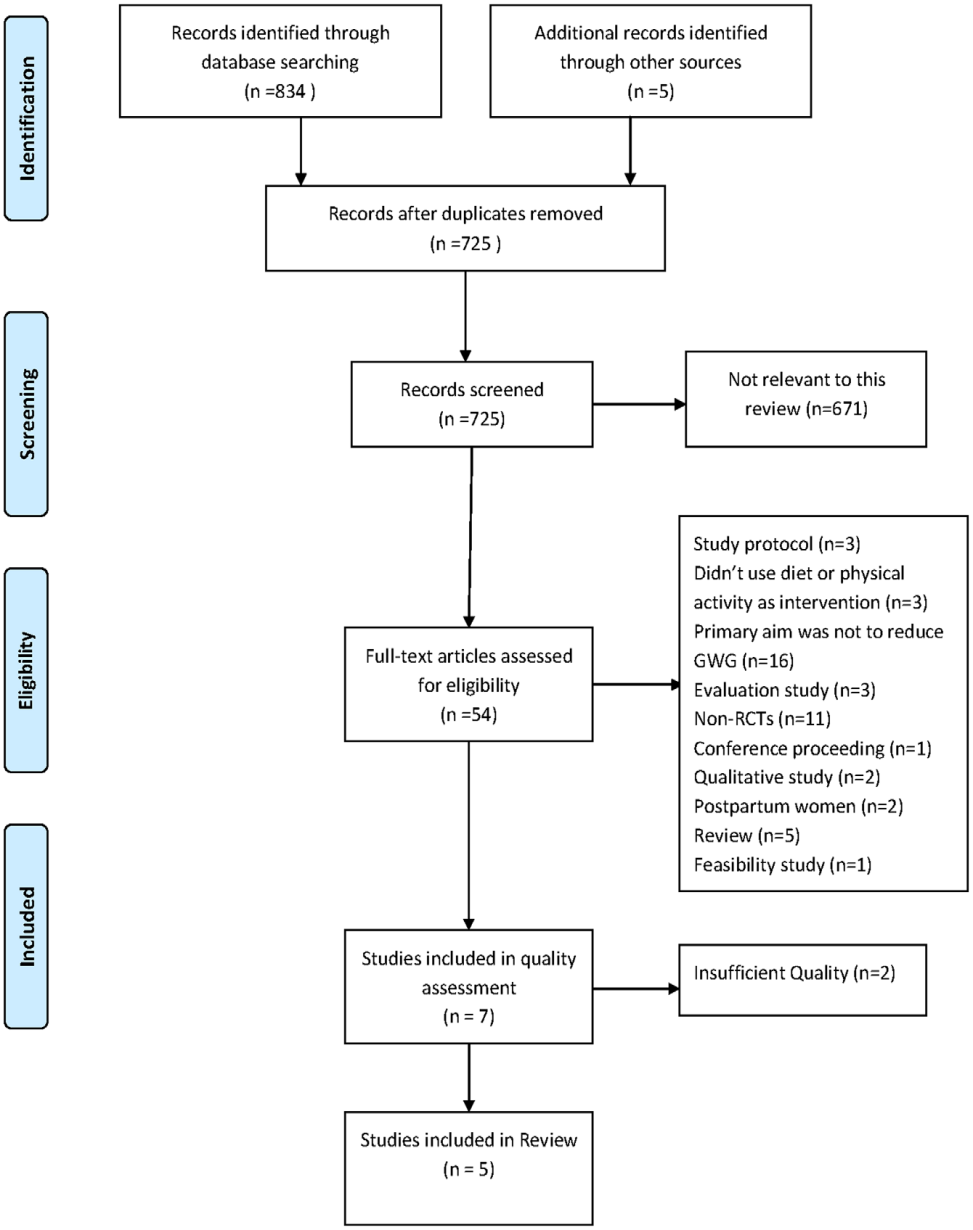
PRISMA Flow diagram of literature search for weight management interventions in pregnancy. The PRISMA flow diagram depicts the flow of information throughout the different phases of this systematic review. It includes the number of records identified, included and excluded and the reasons for exclusions.

Literature searches were conducted bi-weekly from November 2010 to April 2011 using the following databases: Medline (1948-present), Embase (1980-present), British Nursing Index (1994-present), CINHAL, Cochrane-Central Library, PubMed-National Library of Medicine and PsycINFO (1806-present). All databases were searched from inception to ensure that this type of review had not already been completed. The following search terms were used in different combinations: pregnancy, weight control, weight gain, weight change, obesity, obesity prevention, physical activity, exercise, diet, intervention, goal setting. Hand-searches of relevant journals and reference lists along with citation tracking were undertaken to ensure a complete collection of all relevant literature (see figure 1).

### Identification and Data Extraction of Papers

The articles were screened by their titles and studies that did not meet the inclusion criteria were excluded. Two researchers independently reviewed and identified the remaining full text articles according to the pre-determined inclusion and exclusion criteria. Differences between reviewers were resolved through discussion with the research team until consensus was reached. Relevant citations were entered into Review Manager (version 5).

### Stage One: Application of an Extended Quality Assessment Tool

The methodological quality of all RCTs was assessed using the ‘Effective Public Health Practice Project Quality Assessment Tool for Quantitative Studies’ [39]. This tool includes criteria for assessing selection bias, allocation bias, confounders, blinding, data collection methods and withdrawals/dropouts, and has been used in public health interventions and systematic reviews of effectiveness [34], [40]. The quality assessment components of the tool are described in table 1. Adaptations to the quality assessment tool included the following:

**Table 1:** Quality assessment components and ratings for adapted EPHPP instrument.

Components	Strong	Moderate	Weak
**Selection bias**	Very likely to be representative of thetarget population and greater than80% participation rate	Somewhat likely to be representativeof the target population and 60–79%participation rate	All other responses or not stated
**Design** *	RCT design with appropriaterandomisation and concealment ofallocation method described	RCT with appropriate randomisationmethod described, concealment ofallocation was not stated or didnot occur	RCT design, randomisation and concealment of allocation method was inappropriate or not stated
**Confounders**	Controlled for at least 80% ofconfounders	Controlled for 60–79% of confounders	Confounders not controlled for, or not stated
**Blinding**	Blinding of outcome assessor andstudy participants to interventionstatus and/or research question	Blinding of either outcomes assessor or study participants	Outcome assessor and study participants are aware of intervention status and/or research question
**Data Collection Methods**	Tools are valid and reliable	Tools are valid but reliability not described	No evidence of validity or reliability
**Withdrawals and** **dropouts**	Follow up rate of >80% of participants	Follow up rate of 60–79% ofparticipants	Follow-up rate of <60% of participants or withdrawals and dropouts not described
**Analyses** *	Use of ITT analysis stated	-	All other methods or not stated

* Components have been adapted from original tool.

#### Study design score

A limitation of this tool was that all studies described as RCTs scored ‘strong’ for study design regardless of whether studies described the randomisation process or concealment of allocation. If the randomisation process was not described fully or was inappropriate the ‘study design’ component was downgraded from ‘strong’ to ‘moderate’. If studies did not describe an appropriate randomisation method *and* concealment of allocation, the study design was downgraded to ‘weak’.

#### Appropriate analyses score

The use of appropriate analyses was included in the quality assessment tool. The importance of Intention-to-treat (ITT) analysis has been highlighted in previous public health reviews [41] and is important in reducing risk of bias, especially in interventions with high drop-out rates [42]. As a result, studies were scored as ‘strong’ if they used ITT analysis and scored ‘weak’ if they did not.

### Stage Two: Integrity of Intervention

Intervention integrity was incorporated into the original quality assessment tool. The integrity of an intervention is the degree to which that intervention is implemented as planned, and can help determine why the intervention was successful or not [34]. Although ‘intervention integrity’ was not included in the overall score, it is considered to be useful in understanding the outcomes of an intervention [43].

When evaluating the integrity of complex interventions, tables of important qualitative data are frequently used by researchers to explain the context of the quantitative evidence [44]. Often the effectiveness of a program or intervention is evaluated that has not been adequately implemented; this is known as making a type III error [45], [46]. For preventive interventions, integrity data are of particular importance due to the complex environment within which these interventions are implemented, causing various obstacles to completing all components as planned [29],[47]. The integrity and implementation of an intervention was assessed in the second methodological stage of this review in two ways:

#### Assessment of five different dimensions of integrity developed by Dane and Schneider [35]

The five different aspects of intervention integrity assessed in this review included adherence; exposure; quality of delivery; participant responsiveness and program differentiation. Within each of these components several different factors were taken into consideration when extracting integrity data. Table 2, gives a detailed description of each of these five components and associated factors.

**Table 2:** Five aspects of intervention integrity adapted from work of Dane and Schneider [34].

**Adherence**	The extent to which specified components of the intervention were delivered as prescribed.
**Exposure**	An index that included any of the following: (a) intensity of intervention; (b) the frequency and length of each session;(c) average length of intervention, or (d) follow-up.
**Quality of Delivery**	A measure of qualitative aspects of delivery that are not directly related to the implementation of the content of the intervention.This included; (e) leader preparedness and training (leader quality) and (f) leader attitude towards the program.
**Participant responsiveness**	A measure of participant response to components of the intervention, which included: (g) participant retention rates and(h) participant enthusiasm.
**Program Differentiation**	To ensure that the participants in each experimental group received only the planned interventions. Therefore included:(i) co-intervention/contamination and (j) continuity of intervention.

Intervention intensity formed part of the ‘exposure’ component of intervention integrity. Often the intensity of an intervention is highlighted during intervention evaluations, yet interventions of high intensity may not be implemented as planned often leading to a false evaluation of its effectiveness. To assign an intensity rating to intervention and control groups for included studies the following classification system was created based on 5 levels of intensity, where level 1 represented lowest intensity and level 5 represented highest intensity [48].

Level 1 = provision of written materials, brochures or educational leaflets and/or verbal advice on diet, physical activity and weight gain in pregnancy.

Level 2 = one-to-one counselling providing a *standardised* diet and physical activity plan.

Level 3 = one-to-one counselling providing a *standardised* diet and physical activity plan + follow up/regular feedback.

Level 4 = one-to-one counselling providing an *individualised* diet and physical activity plan + follow-up/regular feedback.

Level 5 = level 4+ access to a program/provision of exercise classes and other forms of support.

#### Evidence of goal setting

A theoretical framework can provide a useful structure for evaluating and synthesising evidence on the effect of an intervention [34]. The use of relevant theory in developing complex interventions is more likely to result in an effective intervention than a purely empirical or pragmatic approach [29]. Therefore, understanding the role of theory is essential in the design and evaluation of interventions [28]. Adding to the aspects of goal setting already evaluated using the five dimensions of integrity [35], the theoretical application of goals reported within the studies was further evaluated using a goal-based framework that was developed for the purpose of this review and based on key components of goal setting identified in the existing literature [49]–[51].

An explicit description of how and when the goal[s] was suggested to women was obtained and recorded in relation to five key components of goal setting:

- Purpose goals*-* defined as the overall reason given to individuals for engaging in healthy eating and/or physical activity during pregnancy such as, ‘this will help you return to your pre-pregnancy weight faster’.
- Target goals*-* defined as task-specific guidance given to the individual that has the ability to lead them in achieving the overall purpose goal such as, ‘engaging in moderate intensity exercise 3–5 times a week’.
- Performance Feedback Indicators- defined as the provision of specific/personalised information that enables the individual to assess their progress towards goal attainment such as regular weight monitoring.
- Goal proximity*-* refers to the frequency of the target or sub-goals and the reinforcement schedule associated with the performance feedback.
- Goal framing*-* refers to whether goals are communicated as positive, approach-orientated goals or negative avoidance-orientated goals i.e. a woman may be trying to “achieve optimal GWG” or trying to “avoid gaining excess weight”. Individuals with primarily avoidance-orientated goals are thought to be at a higher risk of emotional distress and anxiety than individuals with primarily approach-orientated goals [52].

A data extraction table (see table 3) outlines the data collected on goal setting components as a source of human motivation associated with the intervention integrity. Two authors reviewed the included studies to assess the nature of the goal setting components reported in each of the interventions. Any disagreements were resolved by discussion and a consensus reached with a third reviewer when required.

**Table 3:** Application of goal-setting components for included studies.

Study	Explicit description of goal theory	Goal proximity	Goal Framing	Purpose goal	Target goals outlined	Performance Feedback Indicators
***Polley et al. *** [***54***]	Goal setting for eating and exercise behaviours	Intervention delivered at regularly scheduled visits [specific number not reported]. Newsletter posted biweekly. Feedback was given after every clinic visit. Participants contacted via phone between clinic visits. Extra counselling sessions for women who exceeded GWG goals ranged from 1–11.	Shortly after recruitment participants were given written and oral information on; appropriate GWG, PA and healthy eatingin pregnancy.	None reported	GWG goal set to correspond with IoM guidelines. Individual counselling sessions included goal setting for eating and PA behaviours. Dietary goals included: decreasing the consumption of high fat foods i.e. fast foods and substituting healthy alternatives. PA goals included; increase walking and developing a more active lifestyle. A stepped care approach was used, where the woman was given increasingly structured behavioural goals at each visit if her weight continued to exceed the recommended levels.	Women were weighed regularly and provided with individual graphs of their weight gain. Those exceeding GWG goals on four consecutive visits were given more intensive intervention including individualised counselling with increasingly structured behavioural goals. Women were contacted by telephone between clinic visits to discuss progress towards the goals set at the previous visit. Self-report measures of dietary intake obtained at recruitment, 30 weeks and 6 weeks postpartum.
						
***Asbee et al. *** [***55***]	None	Participants met with a dietician on the first visit for lifestyle counselling. Feedback on GWG was provided after every routine antenatal appointment [specific number not reported].	On the first visit participants were given information on diet, PA and appropriate GWG.	None reported	GWG goals were set to correspond with IoM guidelines. Dietary goals included recommendations to eat a diet of calorie value divided in a 40% CHO, 30% protein and 30% fat. Exercise goals included advice to engage in moderate intensity exercise at least 3 times per week, preferably 5.	Regular weight monitoring occurred where participants’ weight was measured and charted on an IoM GWG Grid. If GWG was within the IoM guidelines the participant was praised and encouraged to continue their diet and exercise routine. If GWG was not within the IoM guidelines then the participant’s PA and diet routine was reviewed and advised on changes.
***Huang et al. *** [***56***]	Goals were set for personal GWG	Intervention was delivered through 6 counselling sessions; 1 primary session (30–40 mins), 5 booster sessions (28 weeks, 36–38weeks, before hospital discharge, 6 weeks PP and 3 months PP). Feedback on GWG was provided after every clinic visit.	At the first session goals were set for personal GWG and a diet and exercise plan was discussed	None reported	Goals were set for personal GWG (within 10–14 kg range). Individualised dietary and PA education plan was provided based on participants’ baselines information. Examples were provided of a healthy diet and appropriate PA plan. A brochure offered detailed information on weight management goals, ideal body weight, diet and PA.	Women were sent a personalised graph of their GWG. At each booster session participants submitted 3 day records of their diet and self-monitored PA. Women were informed of whether their weight changes were within the appropriate ranges and encouraged to maintain a healthy lifestyle. Those whose weight exceeded GWG goals were given an additional assessment of current diet and PA, problem solving and goal setting for diet and PA behaviours.
***Phelan et al. *** [***57***]	Specific goals were provided	Intervention was delivered through one counselling session at the onset of treatment. Feedback on GWG was provided at every clinic visit which typically occurred monthly until 28 weeks, bi weekly for 28–36 weeks, weekly until delivery and at 6 weeks pp. Postcards were mailed weekly. Three×10–15 min supportive phone calls. Participants under or over GWG goal in any 1 month interval had additional brief supportive phone calls (2 calls/month) and provided with structured meal plans and more specific goals.	At the beginning of the intervention appropriate GWG, PA and dietary goals were discussed.	None reported	GWG goals were set to correspond with IoM guidelines. Dietary goals included the reduction of high fat foods and aiming for calorie goals based on 20 kcal/kg. PA goals included 30 min of walking on most days of the week. Automated postcards that prompted healthy eating and exercise habits were mailed weekly. Supportive phone calls were also provided.	After each clinic visit, women were sent personalized graphs of their weight gains with feedback. Women who were over or under weight gain guidelines during any 1 month interval received additional brief, supportive phone calls. BW scales, food records and pedometers were provided to promote adherence to daily self-monitoring.
***Wolff et al. *** [***58***]	None	Intervention was delivered through 10 counselling sessions lasting 1 hour each. Food records were obtained at inclusion, 27 and 36 weeks and feedback was given.	Not reported	None reported	Restrict GWG to 6–7 kg. Healthy eating goals were set according to the official Danish dietary recommendations (fat intake: max 30 E %, protein intake: 15–20 E%, carbohydrate intake: 50–55 E%). Energy intake was based on individually estimated requirements.	Food records were used as a self-monitoring tool to identify unhealthy eating patterns and give individualised suggestions for improvement. Participants were weighed at inclusion, 27 and 36 weeks gestation [not specified whether feedback was given].

It is important to recognise that the dimensions of integrity overlap with goal setting. For example ‘exposure’ as a dimension of intervention integrity, has the potential for motivational effect in that a person who is exposed more frequently to an intervention, may have a greater probability of receiving more frequent and motivating feedback. While it is not possible within the limitations of this review to report the different aspects of motivational goal setting as an integrated component of intervention integrity, they should be considered alongside each other.

## Results

Results were analysed and reported in relation to the 3 levels; namely quality assessment, intervention integrity and implementation of goal setting components.

### Results of Search

Figure 1 identifies the procedure adopted to identify relevant studies for this review. Overall, a total of 839 articles were identified through database searching and hand searching methods; 114 duplicates were removed. From the remaining 725 unique articles 671 did not explicitly address the subject under review. Therefore, 54 full text articles were assessed for eligibility. Forty seven articles did not meet eligibility criteria and were excluded at this stage. Seven studies were assessed for methodological quality and 2 studies were excluded due to insufficient quality [53], [54]. This resulted in a total of 5 studies being selected for inclusion in this review.

### Characteristics of Included Studies

Table 4 provides a more detailed description of the characteristics of included studies.

**Table 4:** Characteristics of included studies.

Study	Participants	Intervention	Comparison	Outcome measures	Results/Conclusion
***Polley et al. *** [***54***]	120 pregnantwomen at<20 weeksgestation	Single, standardised, one-to-onediet and lifestyle counsellingsessions. Weekly mailed writtenmaterial. Regular weightmonitoring. Supportivetelephone calls.	StandardAntenatal Care	GWG above IoM guidelines	Intervention group had a significantly lower number of NW women exceeding IoM guidelines vs. control group. No significant improvements in GWG for OW/OB women.
***Asbee et al. *** [***55***]	100 pregnantwomen, at6–16 weeksgestation	One-to-one, standardiseddietary and lifestyle counselling.Instructed to engage inmoderate-intensity exerciseat least 3 times per week.Regular weight monitoring.	StandardAntenatal Care	GWG within the IoM guidelines.	Intervention group gain significantly less weight than the control group. No statistically significant differences between groups in the adherence to IoM guidelines.
***Huang et al. *** [***56***]	189 pregnantwomen<16 weeksgestation	Six, individualised, one-to-onediet and lifestyle counsellingsessions. Regular weightmonitoring. Researcher-preparedbrochure.	StandardAntenatal Care	Body weight changes.Psycho-social variablessuch as; health-promoting behaviour,self-efficacy, bodyimage, depression,social support.	All outcome indicators were significantly better for intervention group vs. control group.
***Phelan et al. *** [***57***]	401 pregnantwomen between10 and 16 weeksgestation	Single, standardised, one-to-onediet and lifestyle counsellingsession. Weekly mailed materials.Regular weight monitoring.Supportive telephone calls.	StandardAntenatal Care	GWG above IoM guidelines	Intervention group had a significantly lower number of NW women exceeding IoM guidelines vs. control group. No significant improvements in GWG for OW/OB women.
***Wolff et al. *** [***58***]	50 obese pregnantwomen at∼15 weeksgestation	Ten, individualized, one-to-onedietary counselling sessionsbased on restrictions onenergy intake.	Standardantenatal care	Weightdevelopment	Intervention group gained significantly less weight than the control group.

Abbreviations: GWG, gestational weight gain; IoM, Institute of Medicine; NW, normal weight; OW, overweight; OB, obese.

### Participants

This review contains studies that included a total of 971 pregnant women (Polley et al. [55], n = 120; Asbee et al. [56], n = 144; Huang et al. [57], n = 240; Phelan et al. [58], n = 401; Wolff et al. [59], n = 66). The overall mean age across the studies was 28 years. The mean pre-pregnancy BMI for participants ranged from 21.0 to 34.7 with an overall mean BMI of 26 across the studies. The study by Wolff et al. [59] recruited obese pregnant women only. Of the studies that reported parity, the number of primiparous participants ranged from 38% to 48% with an overall mean of 44%. Polley et al. [55] targeted the intervention program at socio-economically deprived pregnant women.

### Intervention

Four studies tested the effects of combined diet and physical activity based interventions [55]–[58] and one study tested the effects of modification to diet only [59]. The main intervention strategy used by all studies was one-to-one diet and lifestyle counselling. Other intervention components involved supportive telephone calls to discuss progress in reaching set goals and the provision of written educational materials including brochures, newsletters and cards [55], [57], [58].

### Comparisons

All studies described control groups as receiving standard prenatal care. However, none of the studies specified in detail what constituted ‘standard prenatal care’.

### Outcomes

#### Primary outcome

Weight-related outcome measures for the 5 studies reviewed included: proportion of women whose GWG was within or above the IoM guidelines [60], BMI and body weight changes. Three studies reported significantly lower GWG in the intervention group compared to the control group for normal weight, overweight and obese women [56], [57], [59]. Two studies reported a significantly lower weight gain in the intervention group when compared to the control group for normal weight women only [55], [58]. On follow-up Huang et al. [57] and Phelan et al. [58] found that weight retention was significantly lower in the intervention group at 6 months postpartum.

#### Secondary outcomes

Psycho-social outcomes: The study by Huang et al. [57] used self-efficacy as a motivational measurement outcome; the study concluded that women in the intervention group had significantly better scores for self-efficacy. In addition, scores for self image increased, while lower levels of depression were noted in the intervention group when compared with the control group. Other psychosocial outcomes reported were: health-promoting behaviours and social support. No other studies reported the use of such psycho-social outcome measures.

Diet and Physical activity behaviours: Two studies [55], [59] reported using diet and physical activity as outcome measures. Polley et al. [55] found that neither diet nor exercise levels were found to be related to the amount of weight gained during pregnancy. However, women who received the intervention by Wolff et al. [59] reported successfully limiting their energy intake and following dietary instructions for the recommended macronutrient composition of the diet.

### Level 1: Quality Assessment

Table 5 details the 5 RCTs scored for methodological quality. Only one trial was considered to be of high methodological quality [58] and four studies were considered to be of moderate methodological quality [55], [57]–[59].

**Table 5:** Quality assessment scores for included studies.

Study	Selection Bias	Allocation Bias	Confounders	Blinding	Data Collection Methods	Withdrawals/Dropouts	Analyses	Global Rating
***Polley et al. *** [***54***]	MODERATE	MODERATE	MODERATE	MODERATE	WEAK	MODERATE	STRONG	MODERATE
***Asbee et al. *** [***55***]	MODERATE	STRONG	STRONG	MODERATE	MODERATE	MODERATE	WEAK	MODERATE
***Huang et al. *** [***56***]	MODERATE	STRONG	STRONG	MODERATE	MODERATE	MODERATE	WEAK	MODERATE
***Phelan et al. *** [***57***]	MODERATE	STRONG	STRONG	MODERATE	STRONG	STRONG	STRONG	STRONG
***Wolff et al. *** [***58***]	MODERATE	MODERATE	STRONG	MODERATE	MODERATE	MODERATE	MODERATE	MODERATE

#### Selection bias

Recruitment of participants was conducted in obstetric clinics and Healthy Start prenatal classes. Even if the sample collected were ‘somewhat’ likely to be representative of the population, bias may have been introduced in that women who are likely to attend a Healthy Start programme, are also those who are more health conscious and may be more highly motivated to achieve.

#### Allocation bias

Allocation concealment was addressed in only two studies [56], [58], both of which used concealed opaque envelopes.

#### Confounders

Most studies addressed confounders even if only a small number. Studies received a strong rating for the confounder item as long as there were no significant differences between groups at baseline.

#### Blinding

Due to the nature of these interventions, blinding of participants can be difficult and therefore is unlikely to have occurred. Only two of the studies clearly reported blinding outcome assessors or personnel to group membership [57], [58].

#### Data collection methods

Only two studies provided sufficient information to assess the validity and reliability of weight measurements [58], [59]. Self-reported weights were used frequently to calculate women’s pre-pregnancy BMI.

#### Withdrawals/dropouts

In all studies, authors described both the numbers and reasons for withdrawals and dropouts.

#### Analyses

Only two of the five studies reported using ITT analysis [55], [58] and one study reported using per protocol analysis [57].

### Level 2: Five Dimensions of Intervention Integrity [35]

Table 6 describes integrity data collected for each included study.

**Table 6:** Integrity of Intervention data for included studies.

Study												
	Adherence	Exposure				Quality of Delivery			Participant Responsiveness		Program Differentiation	
		*a*	*b*	*c*	*d*	*e*		*f*	*g*	*h*	*i*	*j*
***Polley et al. *** [***54***]	*	**Control group:** level 1 **Intervention group:** Level 3/4	Unclear- regularlyscheduled clinic visitswith phone calls inbetween visits.	29 weeks	8 weekspostpartum	Masters/doctoral prepared staff with training in nutrition or clinical psychology.	*		62%	*	Unlikely	Low
***Asbee et al. *** [***55***]	*	**Control group:** Level 1 **Intervention group:** Level 3	1 counselling session at time of enrolment.	23 weeks	Delivery	Registered dieticians	*		69%	*	Unlikely	Low
***Huang et al. *** [***56***]	*	**Control group:** Level 1 **Intervention group:** Level 4	6×30–40 minscounselling sessions.	47 weeks	6 monthspost partum	Masters-prepared nurse with training in nutrition and physical fitness.	*		78%	*	Unlikely	Low
***Phelan et al. *** [***57***]	*	**Control group:** Level 1 **Intervention group:** Level 4	1 counselling sessionand 3 supportive phonecalls. Phone calls lasted approximately10–15 mins.	49 weeks	6 monthspost partum	*	*		82%	*	Unlikely	Low
***Wolff et al. *** [***58***]	*	**Control group:** level 1 **Intervention group:** Level 4	10×1 hour dietary counselling sessions	22 weeks	4 weeksPostpartum	Trained dietician	*		62%	Evidence that women found the intervention too time consuming (13 drop outs early in the study)	Unlikely	Low

Abbreviations: *No data was available for included study, ***a*** *Intensity of Intervention,* ***b*** *Number., frequency and length of sessions,* ***c*** *Average length of intervention [presuming full term delivery at 37 weeks],* ***d*** *Follow up,* ***e*** *Leader quality,* ***f*** *Leader attitude,* ***g*** *Compliance[measured as retention rate],* ***h*** *Participant enthusiasm,* ***i*** *Contamination/co intervention,* ***j*** *Continuity of intervention.*

#### Adherence

Adherence measures are usually obtained through observational procedures. Lack of process evaluation studies and feedback related to the integrity of the implementation made it difficult to determine whether specified components of each intervention were delivered as prescribed.

#### Exposure

Intervention intensity varied across studies. Two studies used individualised diet and physical activity regimes [57], [59]. Three studies used standardised counselling providing participants with general healthy eating and physical activity goals relevant to all pregnant women [55], [56], [58]. Interventions by Polley et al. [55] and Phelan et al. [58] were classified as level 3/4 intensity. When a woman exceeded weight gain guidelines on four consecutive appointments intervention intensity increased through the re-setting of more specific target goals, and the provision of *individualised* diet and lifestyle counselling as opposed to standardised counselling.

The number of sessions implemented varied in each study. The most time intensive interventions were those by Wolff et al. [59] and Huang et al. [57]. The study by Polley et al. [55] did not state how many sessions were implemented.

Asbee et al. [56] followed women through until delivery and the remaining studies followed women into the postpartum period with assessment at 4 weeks [58], 8 weeks [54] and 6 months [56], [57] postpartum. The study by Huang et al. [57] was the only study that continued to implement the intervention during the postpartum period.

#### Quality of delivery

Leader quality was unclear in the study by Phelan et al. [58]. Lack of process evaluations meant that no information was provided for leader enthusiasm or attitude. Asbee et al. [56] was the only study that reported education and training of healthcare professionals on the study goals and protocol to standardise counselling techniques.

#### Participant responsiveness

Polley et al. [55] had the lowest retention rate of 62% at 6 weeks postpartum. At the time of delivery the retention rate was 92%, resulting in a participant loss of 30% from delivery to 6 weeks postpartum.

Trials by Huang et al. [57] and Phelan et al. [58] lasted an average of 47 and 49 weeks and had retention rates of 78% and 82% respectively. This was considerably higher than the other three studies that lasted an average of 23 weeks with an average retention rate of 69%.

Feedback from participants on the intervention was not reported in four of the studies. Only Wolff et al. [59] reported that women found the intervention time consuming and that this had contributed to a high number of dropouts early in the trial.

#### Program differentiation

No studies reported having measures in place to ensure that all participants received the same intervention. Only one study discussed the limitations of being unable to strictly control the counselling that actually took place at each visit [56]. Counselling sessions in all studies were delivered by dieticians or masters/doctoral level nurses or staff. As other health care professionals involved in delivering antenatal care did not receive training, it may be a possibility that women received inconsistent information and advice.

For most of the included studies, recruitment and delivery of the intervention occurred at the same study site. The only exception was the intervention by Asbee et al. [56] which conducted the study outside of the clinic where recruitment took place. For those studies where one site was used, contamination bias is possible; yet most studies did not report methods to reduce contamination bias or co-intervention. Despite this, interventions consisted mainly of one-to-one counselling sessions with staff who did not routinely deliver antenatal care, therefore contamination is unlikely.

### Level 3: Application of Goal Setting Components within the Interventions

Table 3 details the goal setting components incorporated into the intervention structures. In relation to the direct application of motivational theory as a means of guiding the development and application of the intervention, only one study by Phelan et al. stated explicitly the application of Social Learning Theory [61]. However, the use of goal setting was evident in all of the included studies, and was explicitly stated by Polley et al., Huang et al. and Phelan et al. [55], [57], [58].

#### Purpose goals

None of the included studies reported providing women with an overall purpose goal or reason as to why they should manage their weight during pregnancy.

#### Target goals

All studies outlined specific target goals as part of the intervention. Each study had an established weight gain goal for each participant corresponding with specific weight gain guidelines. Four studies based women’s target goals on the IoM guidelines (1990) [55], [56], [58], [59] and Huang et al. [57] used the weight gain range recommended by the Department of Health in Taiwan (10–14 kg).

Other stated target goals focused on improving diet and physical activity levels to promote optimal weight gain. The most common types of diet and exercise target goals were provided through diet and exercise plans.

To facilitate goal achievement the following was used: standardised and individualised diet and exercise regimes, behavioural modification techniques, self-monitoring, problem-solving and the provision of additional support when required. Two studies explicitly stated the use of problem-solving as a motivational strategy within their one-to-one counselling sessions [55], [57].

#### Feedback performance indicators

All five studies used multiple performance feedback indicators including:

- Weight monitoring where women were weighed regularly throughout the study period
- Visualisation of their personal success or failure using weight gain graphs
- Verbal feedback
- Self-monitoring of diet and physical activity through the provision of body weight scales, food records and pedometers [55], [57]–[59]

#### Goal framing

In all studies, optimal weight target goals were transferred to the women at the first one-to-one counselling session though discussion of appropriate weight gains during pregnancy. There is insufficient information provided by the authors to conclude whether the language used to communicate the suggested goal structure was approach-orientated or avoidance-orientated. However some reference to avoidance-orientation is provided, for example Wolff et al. [59] does report the identification of unhealthy eating patterns as behaviour to be avoided.

#### Goal proximity

As shown in table 3 the frequency with which the target goals are set and feedback provided varied considerably between studies. It is important to note that in two of the five studies reviewed, the frequency of reinforcement and feedback increased when women failed to reach specific target goals [55], [58]. Telephone messaging was used as a follow-up and source of encouragement for women who required additional intensive support [55], [58]. While the potential for motivation via this type of messaging exists, the authors did not describe the content or motivational nature of telephone calls/postcards [62].In the studies by Asbee et al. [56] and Huang et al. [57] a review of participants’ current diet and physical activity regimes was provided for women who were not within IoM guidelines. As already pointed out, Wolff et al. [59] used food records to identify unhealthy eating patterns and provide suggestions for improvement.

## Discussion

The aim of this review was to explore the use of goal setting within healthy lifestyle interventions for the prevention of excess GWG. The results of this review have concluded that interventions based on goal setting can be useful for helping women to achieve optimal weight gain during pregnancy. However, due to the important limitations in methodological quality and lack of homogeneity, especially in relation to how goals were set and supported, it is not possible to identify which aspects of goal setting are most successful in facilitating optimal GWG.

This review identified seven RCTs that involved modification to diet and/or physical activity levels, with the primary aim to prevent excessive GWG. Of the seven studies, only five were found to be of sufficient methodological quality to be included in this review. A comparison between studies was difficult due to differences in the study population (e.g. number of participants, low-income women), length of follow-up, design (e.g. ITT) and intervention given (type, exposure, intervention integrity and goal setting). The heterogeneity of the type of interventions applied has been confirmed through analyses performed in previous reviews in this area [15]–[17].

Several methodological limitations were found in the studies reviewed that could introduce potential bias and compromise the quality of trials. All studies reviewed were described as randomised controlled trials, however, details of the randomisation method or allocation concealment were often not described or described in insufficient detail. Lack of description of randomisation method does not necessarily mean that the study randomisation protocol was ‘weak’. Therefore, this review may have overlooked other high quality RCTs that could have informed the results of this study and provided valuable information on interventions designed to prevent excess GWG.

Very few studies addressed blinding and only three studies reported blinding outcome assessors/personnel. Although weight-related outcomes were fairly objective and lack of blinding is unlikely to introduce a high risk of bias, there is still a potential for bias that could be avoided in future studies. Despite high dropout rates, only two studies reported using ITT analysis. Ideally ITT analyses should be performed in all studies as they are thought to reduce bias caused by the loss of participants [42].

### Integrity of Interventions

The lack of process evaluations for the reported studies made it difficult to adequately assess intervention integrity. Complexity of the interventions and the lack of reporting of the implementation process were overlooked in all of these studies. This made it difficult to determine whether specified components of each intervention were delivered as prescribed and may indicate inadequate implementation. Process evaluations are essential for identifying the key components of an intervention that are effective [44]. The reporting of successful outcomes is of limited usefulness if interventions are unable to identify what factors were responsible for the positive outcomes [46]. Future studies would benefit from reporting feedback on the five aspects of intervention integrity [35].

Retention rates were acceptable and similar across included studies with an average retention rate of 73%. This is comparable with other weight loss and lifestyle interventions that reported retention rates of 68% [63], [64]. In this review, the study by Phelan et al. [58] had higher than average retention rates at 82%. This is most likely because participants were paid $25 for attending each assessment. No qualitative information on participant enthusiasm was provided in any of the studies, however high retention rates (76% and 78%) in the trial by Wolff et al. [59] and Huang et al. [57] may suggest that these interventions were satisfactory for pregnant women. Participants previously reported reasons for non-completion have been that interventions were too time consuming, suggesting that lifestyle interventions may be difficult for pregnant women to commit to, and consequently may only be suited to more motivated participants [54], [59].

None of the studies reviewed reported having measures in place to ensure continuity of intervention. All of the studies were delivered by more than one interventionist, therefore the content and delivery of information in the counselling sessions may have differed between individuals, introducing bias. Only one study [56] addressed the limitations of being unable to strictly control the counselling that took place at each visit. However, generalisability is substantially weakened with only one interventionist and this could account for another type of bias. Future interventions may benefit from having measures in place to ensure continuity throughout the intervention. For example, if multiple interventionists are used then there should be a training component, and some evaluation of how the goal structures were supported to assist in the standardisation of counselling. Otherwise integrity and implementation cannot be accounted for.

### Goal Setting

It is unclear as to what role theory plays in the effectiveness of an intervention [16]. Within this review only one included study reported the use of Social Learning Theory in intervention design [58]. Various degrees of goal setting components were evident in all studies. None of the included studies reported an overall purpose goal or reason for managing weight during pregnancy. This may be due to lack of information provided in the papers or may highlight a more serious theoretical deficit within these interventions.

Qualitative research suggests that pregnant women may not be aware of the importance of weight gain restriction nor the benefits of eating healthily or exercising during pregnancy [65]–[67]. Gardner et al. [16] recommends the targeting of attitudinal and motivational change through the provision of information about the adverse maternal and perinatal outcomes associated with excess GWG. Future studies need to ensure that women are given a clear purpose goal related to the importance of optimal weight gain in pregnancy in order to facilitate behaviour change and assist in the achievement of target goals. In addition, individualised purpose goals may help improve motivation for example, ‘helping you to return to your pre-pregnancy weight faster’ may motivate a more weight-conscious individual, while reducing risk factors associated with birth might have a stronger motivational effect for women who are considered overweight or obese.

Only two studies [57], [59] provided women with individualised target goals at the very beginning of the intervention as opposed to the application of standardised goal setting offered to all women. However, Polley et al. [55] and Phelan et al. [58] did provide women with individualised goals after they had exceeded weight gain targets at four consecutive visits. The failure to attain the goals set in overweight and obese individuals within these two interventions suggests that individualised goal setting may need further theoretical design. An evaluation study by Claesson et al. [68] reported that pregnant women must be actively involved in setting their own goals and given continuous feedback and reinforcement over the long term to prevent excessive weight gain in pregnancy. Due to the lack of information reported in the studies it was difficult to adequately assess goal proximity and goal framing.

### Primary Outcomes

All included interventions reported significantly lower GWG in the intervention group compared to control group [55]–[59]. However, the study by Asbee et al. [56] is limited by the fact that predetermined power was not achieved. Interventions by Wolff et al. [59] and Huang et al. [57] were successful in restricting weight gain in *all* women. High retention rates and successful outcomes suggest that these interventions may be feasible for facilitating women in achieving optimal GWG. Reasons why these interventions were successful are unclear and could be due to a number of factors such as higher intensity and exposure including greater goal proximity, provision of individualised goal setting (rather than standardised goal setting) or implementation of the intervention into the postpartum period [57]. The causal links between the components of the interventions and successful goal attainment cannot be determined based on the reported information.

Nevertheless, the studies reviewed provide some evidence that these types of interventions can facilitate women in achieving optimal GWG, so preventing long term obesity in women. However, overweight and obese women may require more theoretically designed interventions. A review by Dodd et al. [21] highlighted an uncertainty about the optimal intensity of diet and lifestyle interventions for overweight and obese women and suggested that more intensive counselling programmes *may* be more effective in this population.

### Secondary Outcomes

The importance of relevant theory in the design and implementation of complex interventions has been highlighted in guidelines from the MRC [29]. Only one study in this review reported the use of psycho-social outcome measures including, self-efficacy, body image, depression and social support [57]. A recent review by Skouteris et al. [18] emphasised the importance of targeting psychological factors as well as behavioural factors in relation to diet and physical activity behaviour in order to prevent maternal obesity and maintain weight loss postpartum. Lack of attention to psychological factors such as self-efficacy, motivation and mood within these types of interventions have been highlighted in other reviews and may be part of the reason for limited success [26], [69].

The importance of addressing psychological factors for long term weight maintenance has been further supported by research in non-pregnant women [70], [71]. Despite the need to make women aware of the risks of excess GWG, growing evidence suggests that increasing knowledge alone is not enough to produce substantial changes in healthy lifestyle behaviours. In fact, providing education, information and advice is merely a first step in the process of behaviour change [72]. Whilst knowledge has a major part to play, pregnant women’s confidence to achieve personal control over diet and exercise can be limited by factors such as fatigue, nausea and physical discomfort [73], [74]. Helping pregnant women to overcome these motivational barriers could increase women’s confidence to perform these behaviours. Future interventions may be more successful in helping women adopt these healthy lifestyle behaviours by using a more comprehensive goal setting approach that takes into account the cognitive, emotional and behavioural factors related to achievement.

### Conclusions

Due to the important limitations in methodological quality and lack of homogeneity between intervention trials it was difficult to determine which aspects of goal setting would be most successful in helping prevent excess GWG.

Interventions based on goal setting can be useful for facilitating women in achieving optimal GWG, however, interventions based on individualised goal setting may be more effective for women who are already overweight or obese.

The lack of process evaluations and integrity data make it difficult to determine whether non-significant results are due to a poorly designed intervention or an incomplete delivery of the specified components [35]. Due to the complexity of public health interventions, future interventions should make process evaluations a critical part of intervention delivery.

Among interventions reporting positive results, a combination of individualised diet and physical activity plans, self-monitoring and performance feedback indicators were described as active components.

### Implications for Future Research

The methodological and theoretical limitations identified in many of these studies make it difficult to determine why some interventions were more successful than others and should be addressed in future work. Future studies should be based on theoretical frameworks such as goal setting which has been reported as being effective in changing lifestyle behaviours. The importance of developing and implementing interventions that are theoretically-designed to target women’s psychological needs as well as their emotional and physical needs must be underlined. In turn, this has implications for the use of motivational measurement outcomes (e.g. self-efficacy) alongside objective outcomes (e.g. GWG) as a better representation of the effectiveness of weight management interventions during pregnancy.

## Supporting Information

Figure S1 **PRISMA 2009 Checklist.** PRISMA checklist contains 27 checklist items relevant to the content of a systematic review and meta-analysis, which include the title, abstract, methods, results, discussion and funding.
(TIF)
