# Supplementary figures and images for: A Systematic Review Investigating Healthy Lifestyle Interventions Incorporating Goal Setting Strategies for Preventing Excess Gestational Weight Gain
Source: PLoS One. 2012 Jul 5;7(7):e39503. doi: 10.1371/journal.pone.0039503 (PMC3390339; doi:10.1371/journal.pone.0039503)

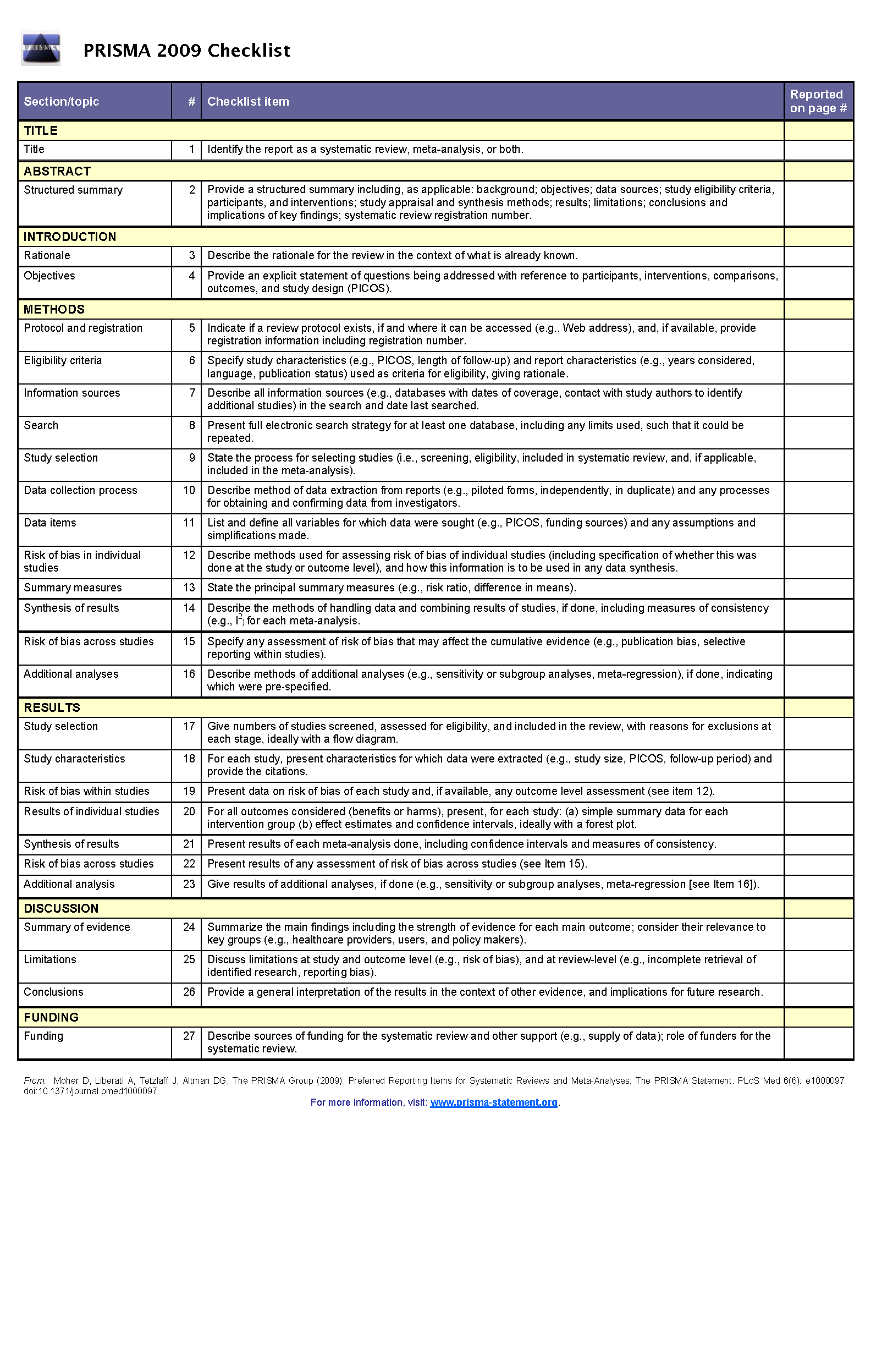

Supplement: Figure S1 — PRISMA 2009 Checklist. PRISMA checklist contains 27 checklist items relevant to the content of a systematic review and meta-analysis, which include the title, abstract, methods, results, discussion and funding. (TIF) [file pone.0039503.s001.tif]
